# Efficacy of Cognitive Behavioral Therapy and Methylphenidate in the Treatment of Attention Deficit Hyperactivity Disorder in Children and Adolescents: A Systematic Review

**DOI:** 10.7759/cureus.32647

**Published:** 2022-12-17

**Authors:** Blessing T Ojinna, Anusha Parisapogu, Mingma L Sherpa, Silpa Choday, Niriksha Ravi, Sheiniz Giva, Vivig Shantha Kumar, Nilasma Shrestha, Hadrian Hoang-Vu Tran, Sai Sri Penumetcha

**Affiliations:** 1 Internal Medicine and Pediatrics, California Institute of Behavioral Neurosciences & Psychology, Fairfield, USA; 2 Infectious Diseases, California Institute of Behavioral Neurosciences & Psychology, Fairfield, USA; 3 Neurology, California Institute of Behavioral Neurosciences & Psychology, Fairfield, USA; 4 Internal Medicine, California Institute of Behavioral Neurosciences & Psychology, Fairfield, USA; 5 Internal Medicine and Neurology, California Institute of Behavioral Neurosciences & Psychology, Fairfield, USA; 6 Neonatology, California Institute of Behavioral Neurosciences & Psychology, Fairfield, USA; 7 Pathology and Internal Medicine, California Institute of Behavioral Neurosciences & Psychology, Fairfield, USA; 8 General Medicine, California Institute of Behavioral Neurosciences & Psychology, Fairfield, USA

**Keywords:** methylphenidate, attention deficit hyperactivity disorder, cognitive behavioral therapy, adolescents, children

## Abstract

The treatment of attention deficit hyperactivity disorder (ADHD) in children and adolescents can be challenging and involve a combination of pharmacologic and non-pharmacological approaches. Using recent literature, we aim to identify the effectiveness of cognitive behavioral therapy (CBT) and methylphenidate (MPH) in reducing the symptoms and improving the quality of life. The investigators conducted a systematic review according to the Preferred Reporting Items for Systematic Reviews and Meta-Analyses (PRISMA) 2020 guidelines. Investigators independently conducted a routine search on PubMed and Google Scholar for articles published within the last five years through July 30, 2022. Fourteen studies were identified as generally good quality but with some limitations. The final analysis included 2098 patients with an age range of three to eighteen. Nine studies reporting the efficacy of MPH in children, adolescents, or both had different formulations and doses. Six studies documenting the effectiveness of CBT had varying sessions, duration per therapy, modality of administration, and participants. The diagnostic assessment measures showed that the parent symptom rating was the highest and appeared in 11 studies, reflecting the burden on the family. In addition, a structured-self-rated questionnaire rating appeared in eight studies, and two diagnostic assessment measures, teacher symptom rating and investigators, appeared in six.

The studies demonstrated significant reductions in the primary symptoms of ADHD at assessment, which led to improved behavioral and functional status with a reduced impact on family and society. Further trials are needed to understand the benefits of CBT and MPH when combined to reduce psychiatry co-morbidities and improve learning and overall quality of life in the long term.

## Introduction and background

Introduction

Attention deficit hyperactivity disorder (ADHD) is a prevalent neurodevelopmental condition in childhood, affecting approximately 4-6% of children and adolescents [1]. The Diagnostic and Statistical Manual of Mental Disorders, 5th Edition (DSM-5) includes ADHD under "Neurodevelopmental Disorders" [1]. ADHD is defined as six or more symptoms of persistent inattention, and/or hyperactivity, and impulsivity, present for six months in two or more settings that interfere with function and are inappropriate for developmental level [2]. Children and adolescents diagnosed with this disorder have difficulty focusing, controlling movements and impulses, and regulating behavior affecting their communication, daily living, and socialization [3].

Boys are two to four times more likely to be diagnosed than girls [4]. In addition, 65% of children diagnosed with ADHD are symptomatic in adulthood, which suggests that the disease is chronic [2]. The etiology of ADHD is multi-factorial and a combination of genetic predisposition and environmental factors, such as low birth weight, prematurity, pregnancy complications, prenatal maternal smoking, intrauterine alcohol exposure, and lead [5]. Family and twin studies have shown a high percentage of heritability with approximately 70% to 80% and substantial overlap between hyperactivity/impulsivity and inattention and with no sex differences in heritability [4]. Although family studies have shown a high heritability and multiple candidate genes that may be involved in the disorder, genome‐wide studies have not yet found a clear association [4].

In addition, over 65% of ADHD patients present with psychiatric comorbidities, including depression, anxiety, and learning disorders, which affect academic performance and family life, with enormous social and economic problems when left untreated [2]. In children younger than six, ADHD is the most common psychiatric reason for referral to a specialist child and adolescent psychiatrist [6]. In addition, parent reports indicate that more than one in ten school-age children (11%, 6.4 million) in the United States have been diagnosed with ADHD by their primary care provider [7].

Clinical practice guidelines recommend a combination of pharmacotherapy and cognitive behavioral therapy (CBT) for treating ADHD in children and adolescents aged six to eighteen years [7]. The psychostimulant, methylphenidate (MPH), has been approved by the United States Food and Drug Administration (FDA) for treating ADHD and is considered a part of the standard of care [7]. ADHD imposes a significant financial burden on families, healthcare systems, and schools [7]. Children and adolescents with ADHD frequently receive special education services, drop out of school, and achieve a much lower rate of post-high school education than their peers [8]. Also, more than 40% of preschoolers diagnosed with ADHD are at risk of suspension, and about 16% are likely to be expelled from school or daycare, compared to only 0.5% of children without ADHD [6]. Consequently, there have been tremendous efforts to develop pharmacological treatments to improve their quality of life and evaluate CBT; for example, cognitive restructuring, thought-stopping, behavioral activation, and exposure techniques to accomplish behavioral management [8].

Research has shown that pharmacological treatments, particularly stimulants and atomoxetine, psychosocial therapies, and their combination are well-established interventions for children and adolescents with ADHD [8]. Treatment options for ADHD in adolescents and children are limited and primarily require prescribing psychostimulant medication as a first-line treatment [3]. Improvements in behavior, attention, interpersonal interactions, cognition, and executive function reinforce stimulant medication's short-term efficacy. MPH and dextroamphetamine are the most prescribed [3]. Nevertheless, the limitations of these medicines (e.g., short-term effects, unknown long-term effects, and adverse effects such as insomnia and anorexia) have led parents and professionals to seek other treatments. Therefore, non-pharmacological interventions that decrease ADHD symptomatology, such as cognitive training, have been considered an excellent potential benefit [3].

The primary care provider roles include diagnosis, medication management, and referrals to other resources, both educational and behavioral [9]. The American Academy of Pediatrics (AAP) has recently updated its 2019 guidelines, providing the basis for managing ADHD. First-line treatment for the preschool age group four to five years is evidence-based parent training in behavior management (PTBM) and teacher-administered behavioral therapy. If there is no improvement, initiating MPH may be considered. For elementary school-aged children six to eleven years, approved medications by FDA, along with PTBM and classroom behavioral interventions, are preferred. For adolescents aged 12-18, FDA-approved medications are the treatment of choice. Evidence-based training interventions and behavioral interventions should also be encouraged [9].

CBT has been described as "a form of psychological therapy that uses cognitive and behavioral techniques to support individuals to change unhelpful behaviors and thought patterns that occur in situations of fear and to learn better ways of coping with them, thereby relieving their symptoms and becoming more effective in their lives" [9,10]. CBT is delivered in a series of structured sessions and is effective for depression, anxiety, eating disorders, and severe mental illness. Results of two studies of adolescents receiving CBT showed improved parental ratings of ADHD symptoms but found little evidence of benefit for functional impairment [9]. On the other hand, MPH, a dopamine and noradrenaline reuptake inhibitor, is the recommended first-line pharmacological treatment for ADHD in many countries, with treatment response rates between 70% and 90%. As stated previously, MPH is one of the most used psychostimulants and has been the most widely studied regarding its efficacy in treating ADHD worldwide [11]. Compliance with medication is a common problem in ADHD treatment. Lack of adherence may lead to reduced effectiveness, increased adverse events, and other consequential issues, hampering the course of pharmacological treatment. Clinicians should routinely assess medication compliance during treatment, and potential problems in adherence should be openly discussed [12]. In deciding whether to initiate pharmacological treatment in school children and adolescents, the severity of ADHD symptoms, as emphasized by clinical guidelines: cases with low and moderate severity "can" while severe cases "should" be offered pharmacological treatment. However, personal factors, for example, the level of suffering, the situation of the patient's family, comorbidities, and global psychosocial functioning, should also be considered [12].

This review aims to systematically evaluate the effectiveness of MPH and CBT, in treating children and adolescents with ADHD using available literature. The study also establishes the most efficacious treatment in the current period.

## Review

Methods

Guidelines

This systematic review of empirical literature was performed in agreement with Preferred Reporting Items for Systematic Reviews and Meta-Analyses (PRISMA) 2020 [13].

Search Databases

Three investigators independently searched PubMed, PubMed Central, Medical Literature Analysis and Retrieval System Online (MEDLINE), and Google Scholar.

Search Strategy

A systematic literature search using boolean logic to perform a database search and boolean search operators "AND" and "OR" were used to connect the keywords. PubMed search for free full-text articles, conducted in humans and published in English from 2017 until July 30, 2022, using medical subject headings (MeSH) terms keywords in the MeSH database are as follows Attention deficit hyperactivity disorder OR ADHD OR Hyperkinetic syndrome OR ("Attention Deficit Disorder with Hyperactivity/drug therapy" [Majr] OR "Attention Deficit Disorder with Hyperactivity/therapy" [Majr]) AND Cognitive behavioral therapy OR Psychotherapy OR Behavioral therapy OR ("Cognitive Behavioral Therapy/methods" [Majr] OR "Cognitive Behavioral Therapy/organization and administration" [Majr] OR "Cognitive Behavioral Therapy/statistics and numerical data" [Majr] OR "Cognitive Behavioral Therapy/trends" [Majr]) AND Methylphenidate OR methylphenidate hydrochloride OR Central nervous system stimulants OR ("Methylphenidate/administration and dosage" [Majr] OR "Methylphenidate/adverse effects" [Majr] OR "Methylphenidate/pharmacology" [Majr] OR "Methylphenidate/therapeutic use" [Majr] OR "Methylphenidate/therapy" [Majr]). We also performed a direct search on Google Scholar using the keywords Attention Deficit Hyperactivity Disorder (ADHD), Hyperkinetic Syndrome, Attention Deficit Disorder With Hyperactivity, Cognitive Behavioural Therapy (CBT), Psychotherapy, Behavioral Therapy, Methylphenidate OR Methylphenidate Hydrochloride, Central Nervous System stimulants, treatment, efficacy, children, and adolescents. The PICO (population, intervention, criteria, outcome) will be outlined (Table 1).

**Table 1:** PICO criteria. P - population, I - intervention, C - control, O - outcome, ADHD - attention deficit hyperactivity disorder

Headings	Definitions
Population	Children and adolescents with ADHD, aged 3-18 years old
Intervention	Cognitive behavioral therapy and Methylphenidate
Control	Healthy controls, placebo, and other treatment options
Outcome	ADHD symptomatology and functional outcomes

Inclusion Criteria

The papers included in this study are within a range of five years, from 2017 to 2022. Only human studies published in English are part of this study. We included randomized control trials, observational studies, systematic reviews, or narrative reviews, including the age group of preschools to adolescents who received MPH and CBT for treating ADHD.

Exclusion Criteria

In our study, the authors excluded studies published before January 2017, studies that were not free full text on PubMed, studies not published in English, and finally, studies in individuals over 18 years of age. Furthermore, we excluded clinical guidelines and letters to the editor.

Study Selection and Quality Check

The studies we shortlisted were then imported into the EndNote software (Clarivate, London, UK) and transferred to the excel sheet, where we removed duplicates. In addition, we performed a manual check to remove any article to which the topic was non-related. Three reviewers independently reviewed papers based on title, keywords, and abstract. In addition, two reviewers thoroughly reviewed full-text articles that passed the initial screening to determine their suitability for inclusion in the systematic review. The quality of the paper included in the study was assessed by the primary author and two secondary authors using the Cochrane risk of bias tool for randomized controlled trials. We interpreted the result from the biases using the agency for healthcare research and quality (AHRQ) standards. Finally, the papers selected were of good quality. Finally, the investigators used the PRISMA diagram to check the quality of systematic reviews for inclusion.

**Figure 1:**
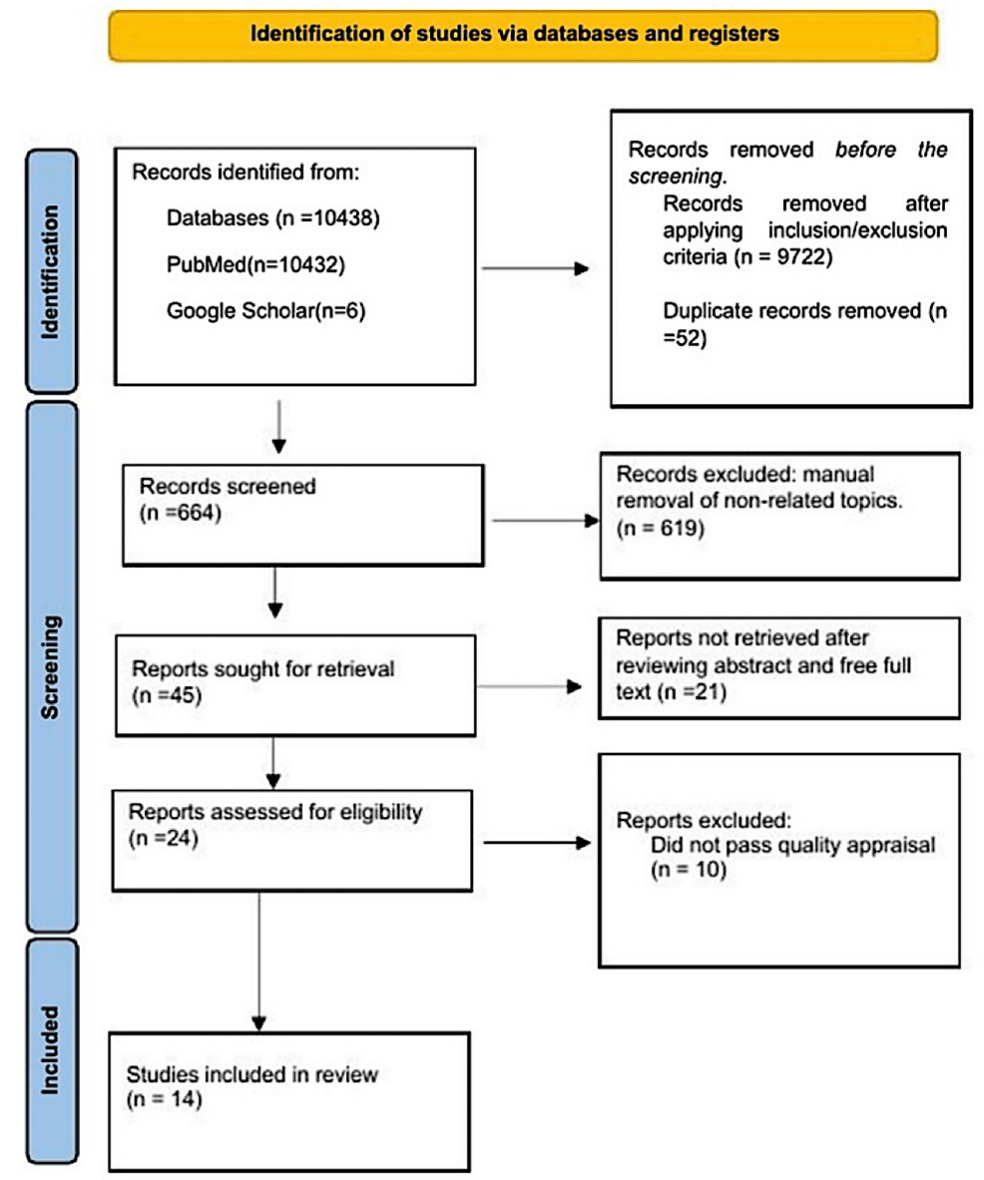
Preferred Reporting Items for Systematic Reviews and Meta-Analysis (PRISMA) 2020 flow diagram depicting the study selection process. Reference [13]

Additionally, we used the scale for assessing narrative review articles (SANRA) checklist to determine if a narrative review was of good quality. Finally, in the event of disagreement, we reached a consensus after discussing it with a fourth author. The PRISMA flow diagram is below in Figure 1 [13].

Results

After a strategic search of various electronic databases, the total number of articles found was 10438 (PubMed - 10432, Google Scholar - six). Records were removed before screening by PubMed filter inclusion criteria = 9722. We removed 52 duplicates with excel. The authors manually screened by title 664 studies and removed 619 articles with a non-related topic. We retrieved 45 records and excluded 21 studies after reviewing abstracts and full text using the eligibility criteria. The investigators identified 24 studies, and 10 did not meet the inclusion criteria after the quality assessment. Finally, we included 14 articles in our review. The PRISMA flow diagram is in the methods section (Figure 1) above. Finally, we outlined the type and number of studies used in this review article (Table 2).

**Table 2:** Type and number of studies included in the analysis.

Type of Study	Number of Studies
Randomized Control Trial	11
Systematic Reviews	2
Narrative Reviews	1

General Study Characteristics

Study participants: The studies included were performed in seven countries; the United States, Taiwan, Norway, Australia, the Netherlands, Denmark, and France [6,7,11,14-24]. The total number of participants recorded was 2,098. The age ranged from 3 to 18 years old, and the mean age observed from the review was 11. However, only one study in this review did not specify the sample size and age range [21]. Therefore, we will explore the essential characteristics of the studies included in this review (Table 3).

**Table 3:** Detailed information about studies included in the review. Detailed information about studies we included in the review. Note: access to the patient during the study determined the sample size#. Diagnostic assessment measures: 1 = parent symptoms rating; 2 = teacher symptom ratings; 3 = structured self-rated questionnaire; 4 = structured parent interview; 5 = performance-based neurological measures; 6 = baseline score as a covariate; 7 = Swanson, Kotkin, Agler, M-Flynn, and Pelham (SKAMP) scale rating; 8 = permanent product measure of performance (PERMP); 9 = investigators; 10 = CGI-S (Clinical Global Impression-Severity, self-report or clinician); 11 = child behavior checklist (CBCL); 12 = child depression inventory and screen for child anxiety. Outcome: improvement in the ADHD core symptoms: a = attention deficit, b = hyperactivity, c = impulsivity/aggressiveness, d = improvement in working memory (accuracy), e = reduction in medication, f = change in symptoms scores on the ADHA rating scale-IV, g = improvement of self-esteem, emotional regulation, and social integration, h = depression/anxiety. DR/ER = delay release/early release, N/A = not applicable, ND = not defined, NFT = neurofeedback is behavioral therapy No. = number, CBT = cognitive behavioral therapy, MPH = methylphenidate

Reference Number	Study Author, Year	Study Design	Country	Diagnostic Assessment Measures	Frequency of Assessment	Sample Size	No. of Reviewed Articles	Age Range (years)	Type of Treatment	Dosage	Duration	Outcome	Efficacy
[14]	Duric et al., 2017	Randomized controlled trial	Norway	1, 2, and 3	3	130, #	NA	11.6 (6-18)	MPH	1 mg/kg/day	6 months	a, b	Stable-throughout the study
[7]	Wigal et al., 2017	Randomized controlled trial	United States	1, 7, 8, 9	Varies hourly	90	NA	6-12	MPH	20-60 mg	7 weeks	a, b, c	yes
[15]	Goode et al., 2018	Systematic Review	United States	10, 11, 12	3^rd^ and 12^th^ month	298	2 for CBT	<17	CBT	ND	12 months	a c, f, h	yes
[16]	Novik et al., 2019	Randomized controlled trial	Norway	1, 2, 3, 9	4	99	NA	14-18	CBT	ND	12 weeks 9 months	f	Limited
[17]	Sciberras et al., 2019	Randomized controlled trial	Australia	1, 3, 4, 9	Baseline, 5^th^, and 12^th ^month	228	NA	8-12	CBT	ND	5 months 12 months	Study protocol	NA
[6]	Childress et al., 2019	Randomized controlled trial	United States	1, 3, 9	ND	119	NA	4-6	MPH	10-40 mg varied	14-16 weeks 12 months	a, b, c	Yes
[11]	Huang et al., 2020	Randomized controlled trial	Taiwan	1, 2, 9	3	100	NA	6-18	MPH	22, 33, and 44 mg	10-12 weeks	a, b, c	Yes
[18]	Coles et al., 2020	Randomized controlled trial	United States	1, 2, 3	9	127	NA	5-13	CBT MPH	ND varied	9 weeks	e	NA
[19]	Wigal et al., 2020	Systematic and clinical review	United States	Mainly 1 and 2, varied	ND	255 79 varied	NA	4-7 3-4 varied	MPH IR	ND	4 years 2 years Varied	a, b	Short-term
[20]	Rosenau et al., 2021	Randomized controlled trial	Netherlands	1 and 5	ND	94	NA	8-18	MPH	Varied	>2 years	d	uncertain
[21]	Ribeiro et al., 2021	Narrative review	Denmark	ND	ND	varied	24	Varied	MPH	ND	ND	Uncertain	NA
[22]	Wilens et al., 2022	Randomized controlled trial	United States	3, 6	2	163	NA	9.3 (mean)	DR/ER MPH	3.7 mg/kg/day 68.1 mg (mean)	5 weeks	a, b, and c	yes
[23]	Vacher et al., 2022	Randomized controlled trial	France	1, 2, 3, and 4	3	68	NA	7-13	CBT	ND	6 months	c	long-term
[24]	Crouzet et al., 2022	Randomized controlled trial	France	1, 3, 9	3	248	NA	7-15	CBT	ND	5months 8months follow up	a, b, c, d, g	hypothetical

Review of Diagnostic Assessment Measures

Two essential treatment modalities, CBT and MPH, were used to assess the primary efficacy outcome: reduced symptoms and improved function in patients with ADHD. The study outcomes were measured using diagnostic assessment measures, which were different in all studies and were undefined in two studies. The "parent symptom rating," the highest applied diagnostic assessment measure, was used in 11 studies. In contrast, eight studies showed that the "structured-self-rated questionnaire" is the second most used diagnostic assessment measure. The other diagnostic assessment measure, "teacher symptom rating" and "investigators," the third most utilized, appeared in six studies.

Type of Treatment Considered Dosage and Duration

The authors analyzed the treatment type, dose, duration of therapy, frequency of assessment, and patient response to demonstrate whether the administration of CBT and MPH is effective. One study assessed MPH and CBT in the included articles [18]. Six studies centered on CBT [15-18,23,24], while nine researchers centered on MPH administration [6,7,11,14,18-22]. The dosage of MPH was different in each of the nine studies. In addition, the duration of the interventions ranged from five weeks to four years, while the frequency of assessment varied extensively.

Review Findings

Our findings showed an improvement in core ADHD symptoms. We observed that eight studies recorded a reduction in inattention, and seven reported a decrease in hyperactivity and impulsivity/aggressiveness. Two more studies also reported improvements in the change in symptom scores using the ADHD rating scale and improved working memory. Nonetheless, Sciberras et al. [17] randomized control trial study outcomes were undefined, and Ribeiro et al. [21] didn't find enough evidence regarding the benefits of MPH on treatment outcomes.

Discussion

A research review, including 11 random controlled trials, two systematic reviews, and one narrative review (Table 2), was used to assess the effects of either CBT or MPH on core ADHD symptoms and function in children and adolescents. This review differs from previously published reviews, intending to focus on the effect of non-pharmacologic therapy (CBT) and stimulant therapy (MPH) as interventions for treating children and adolescents with diagnosed ADHD. In addition, the author's included the established rating scales used in assessing ADHD symptoms in children, as shown in (Table 4) for better understanding [19].

**Table 4:** Rating scales for assessment of ADHD symptoms in children and adolescents. ADHD - attention deficit hyperactivity disorder; DSM-IV-TR - Diagnostic and Statistical Manual of Mental Disorders, fourth edition, text revisions; DSM-5 - Diagnostic and Statistical Manual of Mental Disorders, fifth edition; RDC-PA - Research Diagnostic Criteria-Preschool Age [19].

Instrument	Age, Years	Type of Scale
Preschool Age Psychiatric Assessment, Egger et al. (2006a)	2-5	The psychiatric symptom/function scale incorporating DSM-IV-TR and RDC-PA diagnostic criteria includes an ADHD module
Conners Parent Rating Scale-Revised, Conners et al. (1998a) Conners Teacher Rating Scale-Revised, Conners, et al. (1998b)	3-17	Validated ADHD-specific rating scale
ADHD Rating Scale–5, parent and teacher versions, DuPaul et al. (2016)	5-17	Validated DSM-5–referenced, ADHD-specific rating scale
Early Childhood Inventory–4, Sprafkin et al. (2002)	3-6	Validated DSM-IV–referenced screening instrument includes subscales for ADHD inattention and hyperactive-impulsive subtypes
Child Behavior Checklist 11⁄2–5, de la Osa et al. (2016)	1.5-5	The validated behavioral symptom checklist includes attention problems and attention-deficit/hyperactivity problems subscale (including hyperactive-impulsive and inattentive types)
Vanderbilt ADHD Teacher and Parent Rating Scales, Wolraich et al. (1998, 2003), and DuPaul et al. (2016)	6-12	Validated DSM–referenced, ADHD-specific rating scale, includes items related to oppositional-defiant/conduct and anxiety/depressive disorders; validated in children 6–12 years of age, but applicable to preschoolers
ADD-H Comprehensive Teacher Rating Scale, Ullmann et al. (1984) and Carlini and Parks (1993)	Kindergarten to Grade 8	The validated Likert response scale with Attention, Hyperactivity, Social Skills, and Oppositional Behavior sections

Several behavioral assessment scales have been specifically designed for this patient population to enable physicians to identify patients with ADHD better while excluding those showing developmentally normal behavior. These include the Conners Parent Rating Scale-Revised (Conners et al. 1998a) and Conners Teacher Rating Scale-Revised (Conners et al. 1998b), the AD/HD Rating Scale-5 parent and teacher versions (DuPaul et al. 2016), the Early Childhood Inventory-4 (Sprafkin et al. 2002), and the Child Behavior Checklist (de la Osa et al. 2016). The Preschool Age Psychiatric Assessment (Egger et al. 2006a) was developed based on the Child and Adolescent Psychiatric Assessment (Angold and Costello 1995) for children two to five years of age. Still, no validation reports in that population have been published to date. The Vanderbilt ADHD Teacher and Parent Rating Scales have been validated in children aged six to twelve but are also applicable to preschool children (Wolraich et al. 1998, 2003; American Academy of Pediatrics 2019). The Conners scales and AD/HD Rating Scale-5 are specific to ADHD behaviors and based on the Diagnostic and Statistical Manual of Mental Disorders (DSM) ADHD criteria. In contrast, the Child Behavior Checklist and Preschool Age Psychiatric Assessment assess a wide range of behaviors and do not reflect criteria for ADHD alone (Conners et al. 1998a, 1998b; Egger et al. 2006a; de la Osa et al. 2016; DuPaul et al. 2016). Physicians must remain cautious in diagnosing ADHD in preschool children, monitoring very young children for the emergence of symptoms and impairments over time [19].

CBT Intervention

The CBT interventions in six studies included in this review had different measures of assessing the effectiveness of the ADHD treatment [15-18,23,24]. The study observed that CBT improved ADHD symptoms (Table 3). However, CBT can be time-limited and resource-intensive. The variations in CBT treatments could be the mode and duration of CBT sessions. Most studies had weekly clinician-led sessions, and the number of sessions varied. The duration of each session also varied between studies lasting an hour long and beyond, and the sessions can be either with individual children/parents [17] or combined [24]. Finally, we will detail the CBT sessions reviewed in our study (Table 5).

**Table 5:** Details of CBT sessions. CBT - cognitive behavioral therapy

Reference Number	Study	Session Led	Number of Sessions	Duration	Participants	
[15]	Goode et al., 2018	Not applicable	Not applicable	Not applicable	Children	
[16]	Novik et al., 2019	Psychologist, psychiatrist, special educator	Weekly sessions for 12 weeks	90 minutes	Adolescents	
[17]	Sciberras et al., 2019	Psychologist	10 sessions over 12 weeks	8 * 1 hour long weekly sessions, 2* 1 hour long fortnightly sessions	Children and parents together	
[18]	Coles et al., 2020	Clinician, teacher	Children daily for three weeks varied no; low high behavioral treatment crossed with MPH. Parents 8 weeks	School year follow up	Children, parents	
[23]	Vacher et al., 2022	Psychologist, Nurse, Educator	Once weekly for 15 weeks, children sessions and 8 parent sessions	1 hour	Children, parents	
[24]	Crouzet et al., 2022	Psychiatrist, Psychologist	Children 16 sessions Parents 16 sessions	75 minutes weekly	Children and parents performed separately	

MPH Treatment

The nine studies included in this review have different formulations of MPH [6,7,11,14,18-22]. Since the advent of MPH in the 1960s, MPH has been the drug of choice for ADHD worldwide and has proven to improve ADHD core symptoms. Pharmacological treatment optimization poses challenges, including careful dose adjustments and the risk of drug abuse during treatment [14]. The study reported marked improvement in ADHD core symptoms six months after treatment completion by parents, teachers, and participants, with significant improvement in inattention [14]. However, the study did not witness a significant improvement in hyperactivity or academic performance [14]. Furthermore, treatment with MPH ERCT (extended-release chewable tablet) showed a significant improvement in ADHD symptoms compared with placebo at two to eight hours post-dose, with a good safety and tolerability profile [7]. In the first multicenter, phase three study of the efficacy and safety of MPH ERCT formulations, the results showed a statistically significant improvement in behavior impairment in children aged six to twelve with ADHD [7].

Additionally, in the first randomized, placebo-controlled trial of an ER MPH formulation for preschool children aged four to six years, doses of up to 40 mg were effective and well tolerated [6]. It is like the known safety profile in older children [6]. Another study, a placebo-controlled crossover trial looking into the clinical efficacy and tolerability of ORADUR-MPH, reported that it significantly reduced symptoms of inattention, hyperactivity, and impulsivity within two weeks of treatment regardless of informants [11]. ORADUR-MPH is efficacious, safe, and well-tolerated for treating ADHD without serious side effects [11].

Limitations

This study has some critical limitations. The major one is the limited number of articles documenting the use of MPH and CBT alone to treat ADHD. Furthermore, a systematic, non-descriptive review of CBT sessions was included [15] and provided limited measures for the discussion. In addition, we included an unresolved research protocol, which can be controversial due to the lack of an evident result. Some studies lacked information on the study characteristics, and we did not contact the authors. This insufficiency in knowledge may have affected the quality of the outcome of the result. Also, our studies used different CBT session approaches and varying formulations and dosages for MPH; therefore, this needs to be considered for overt improvement.

Finally, our search strategy excluded studies published before the last five years and not freely available full text on PubMed. The inclusion of these might have provided more clarity.

## Conclusions

The purpose of this review is to critically evaluate the efficacy of CBT and MPH in treating ADHD in children and adolescents to improve their core symptoms and functional capacity from published literature. The findings from the review of 2098 patients undergoing either or both treatment interventions showed significant reductions in the primary symptoms of ADHD at assessment, which led to improved behavior and functional status with an overall reduced impact on family and society. Additionally, accurate diagnosis by physicians using the rating scales is key to treatment choice. We observed how CBT helps with behavior management and the role of psychologists, parents, and teachers in ensuring effective therapy. Further trials are needed to understand the benefits of CBT and MPH when combined to reduce psychiatry co-morbidities and improve learning and overall quality of life in the long term.

## References

[1] Miklós M, Futó J, Komáromy D, Balázs J (2019). Executive function and attention performance in children with ADHD: effects of medication and comparison with typically developing children. Int J Environ Res Public Health.

[2] Gomez-Sanchez CI, Carballo JJ, Riveiro-Alvarez R (2017). Pharmacogenetics of methylphenidate in childhood attention-deficit/hyperactivity disorder: long-term effects. Sci Rep.

[3] Veloso A, Vicente SG, Filipe MG (2020). Effectiveness of cognitive training for school-aged children and adolescents with attention-deficit/hyperactivity disorder: a systematic review. Front Psychol.

[4] Storebø OJ, Pedersen N, Ramstad E (2018). Methylphenidate for attention deficit hyperactivity disorder (ADHD) in children and adolescents - assessment of adverse events in non-randomised studies. Cochrane Database Syst Rev.

[5] Shirafkan H, Mahmoudi-Gharaei J, Fotouhi A, Mozaffarpur SA, Yaseri M, Hoseini M (2020). Individualizing the dosage of methylphenidate in children with attention deficit hyperactivity disorder. BMC Med Res Methodol.

[6] Childress AC, Kollins SH, Foehl HC, Newcorn JH, Mattingly G, Kupper RJ, Adjei AL (2020). Randomized, double-blind, placebo-controlled, flexible-dose titration study of methylphenidate hydrochloride extended-release capsules (Aptensio XR) in preschool children with attention-deficit/hyperactivity disorder. J Child Adolesc Psychopharmacol.

[7] Wigal SB, Childress A, Berry SA (2017). Efficacy and safety of a chewable methylphenidate extended-release tablet in children with attention-deficit/hyperactivity disorder. J Child Adolesc Psychopharmacol.

[8] Evans SW, Owens JS, Wymbs BT, Ray AR (2018). Evidence-based psychosocial treatments for children and adolescents with attention deficit/hyperactivity disorder. J Clin Child Adolesc Psychol.

[9] Shrestha M, Lautenschleger J, Soares N (2020). Non-pharmacologic management of attention-deficit/hyperactivity disorder in children and adolescents: a review. Transl Pediatr.

[10] Wang HI, Wright B, Tindall L (2022). Cost and effectiveness of one session treatment (OST) for children and young people with specific phobias compared to multi-session cognitive behavioural therapy (CBT): results from a randomised controlled trial. BMC Psychiatry.

[11] Huang YS, Yeh CB, Chen CH, Shang CY, Gau SSF (2021). A randomized, double-blind, placebo-controlled, two-way crossover clinical trial of ORADUR-methylphenidate for treating children and adolescents with attention-deficit/hyperactivity disorder. J Child Adolesc Psychopharmacol.

[12] Mechler K, Banaschewski T, Hohmann S, Häge A (2022). Evidence-based pharmacological treatment options for ADHD in children and adolescents. Pharmacol Ther.

[13] Page MJ, McKenzie JE, Bossuyt PM (2021). The PRISMA 2020 statement: an updated guideline for reporting systematic reviews. BMJ.

[14] Duric NS, Assmus J, Gundersen D, Duric Golos A, Elgen IB (2017). Multimodal treatment in children and adolescents with attention-deficit/hyperactivity disorder: a 6-month follow-up. Nord J Psychiatry.

[15] Goode AP, Coeytaux RR, Maslow GR (2018). Nonpharmacologic treatments for attention-deficit/hyperactivity disorder: a systematic review. Pediatrics.

[16] Nøvik TS, Haugan ALJ, Lydersen S, Thomsen PH, Young S, Sund AM (2020). Cognitive-behavioural group therapy for adolescents with ADHD: study protocol for a randomised controlled trial. BMJ Open.

[17] Sciberras E, Efron D, Patel P (2019). Does the treatment of anxiety in children with attention-deficit/hyperactivity disorder (ADHD) using cognitive behavioral therapy improve child and family outcomes? Protocol for a randomized controlled trial. BMC Psychiatry.

[18] Coles EK, Pelham WE, Fabiano GA (2020). Randomized trial of first-line behavioral intervention to reduce need for medication in children with ADHD. J Clin Child Adolesc Psychol.

[19] Wigal S, Chappell P, Palumbo D, Lubaczewski S, Ramaker S, Abbas R (2020). Diagnosis and treatment options for preschoolers with attention-deficit/hyperactivity disorder. J Child Adolesc Psychopharmacol.

[20] Rosenau PT, Openneer TJC, Matthijssen AFM (2021). Effects of methylphenidate on executive functioning in children and adolescents with ADHD after long-term use: a randomized, placebo-controlled discontinuation study. J Child Psychol Psychiatry.

[21] Pereira Ribeiro J, Arthur EJ, Gluud C, Simonsen E, Storebø OJ (2021). Does methylphenidate work in children and adolescents with attention deficit hyperactivity disorder?. Pediatr Rep.

[22] Wilens TE, Faraone SV, Hammerness PG (2022). Clinically meaningful improvements in early morning and late afternoon/evening functional impairment in children with ADHD treated with delayed-release and extended-release methylphenidate. J Atten Disord.

[23] Vacher C, Romo L, Dereure M, Soler M, Picot MC, Purper-Ouakil D (2022). Efficacy of cognitive behavioral therapy on aggressive behavior in children with attention deficit hyperactivity disorder and emotion dysregulation: study protocol of a randomized controlled trial. Trials.

[24] Crouzet L, Gramond A, Suehs C, Fabbro-Peray P, Abbar M, Lopez-Castroman J (2022). Third-generation cognitive behavioral therapy versus treatment-as-usual for attention deficit and hyperactivity disorder: a multicenter randomized controlled trial. Trials.

